# A modified anterior drawer test for anterior cruciate ligament ruptures

**DOI:** 10.1186/s13018-021-02381-x

**Published:** 2021-04-14

**Authors:** Guang-lei Zhao, Jin-yang Lyu, Chang-quan Liu, Jian-guo Wu, Jun Xia, Gang-yong Huang

**Affiliations:** 1 Department of Orthopaedic Surgery, Huashan Hospital Fudan University, 12th Wulumuqi Middle Road, Jing’an District, Shanghai, China; 2 Department of Orthopaedic Surgery, North Branch of Huashan Hospital Fudan University, 518th Jingpohu Road, Bao’shan District, Shanghai, China

**Keywords:** Physical examination, Anterior cruciate ligament ruptures, Anterior drawer test, Lachman test, Pivot shift test

## Abstract

**Objective:**

This study was aimed to utilize a modified anterior drawer test (MADT) to detect the anterior cruciate ligament (ACL) ruptures and investigate its accuracy compares with three traditional tests.

**Methods:**

Four hundred patients were prospectively enrolled between January 2015 and September 2017 preoperatively to undergo knee arthroscopic surgeries. The MADT, anterior drawer test, Lachman test, and pivot shift test were used in the outpatient clinical setting and were compared statistically for their accuracy in terms of ACL ruptures, with arthroscopic findings as the gold standard.

**Results:**

The prevalence of ACL ruptures in this study was 37.0%. The MADT demonstrated the highest sensitivity (0.89) and accuracy (0.92) among the four tests and had comparable specificity (0.94) and a positive predictive value (0.90) compared with the anterior drawer test, Lachman test, and pivot shift test. The diagnostic odds ratio (DOR) of MADT was 122.92, with other test values of no more than 55.45. The area under the receiver operating characteristic curve (AUC) for the MADT was 0.92 ± 0.01, with a significant difference compared with that for the anterior drawer test (*z* = 17.00, *p* < 0.001), Lachman test (*z* = 9.66, *p* = 0.002), and pivot shift test (z = 16.39, *p* < 0.001). The interobserver reproducibility of the MADT was good, with a kappa coefficient of 0.86.

When diagnosing partial tears of ACL, the MADT was significantly more sensitive than the anterior drawer test (*p* < 0.001), Lachman test (*p* = 0.026), and pivot shift test (*p* = 0.013). The MADT showed similar sensitivity in detecting anteromedial and posterolateral bundle tears (*p* = 0.113) and no difference in diagnosing acute and chronic ACL ruptures (*χ*^2^ = 1.682, *p* = 0.195).

**Conclusions:**

The MADT is also an alternative diagnostic test to detect ACL tear, which is equally superior to the anterior drawer test, Lachman test, and pivot shifting test. It could improve the diagnosis of ACL ruptures combined with other clinical information including injury history, clinical examination, and radiological findings.

**Levels of evidence:**

Level II/observational diagnostic studies

**Trial registration:**

Chinese Clinical Trial Registry. ChiCTR1900022945 /retrospectively registered

**Supplementary Information:**

The online version contains supplementary material available at 10.1186/s13018-021-02381-x.

## Introduction

The anterior cruciate ligament (ACL) is one of the most commonly injured structures of the knee joint [1, 2]. Arthroscopic surgery is the gold standard to diagnose tears of the ACL [3]. Magnetic resonance imaging (MRI) is a good but expensive noninvasive diagnostic tool with 94 to 98% specificity and sensitivity [4–7]. Early detection of an ACL rupture using an accurate physical test is essential to avoid unnecessary additional procedures.

Historically, three widely used physical examinations have been used to diagnose ACL ruptures: the anterior drawer test, Lachman test, and pivot shift test [8–10]. Among them, the time-honored anterior drawer test is the best known and most frequently used, but this test is not sensitive enough to diagnose ACL ruptures in acute injuries compared with chronic injuries. The Lachman test is the most accurate and reliable method to diagnose an ACL rupture, and the pivot shift test is believed to be the most specific but the least sensitive of the three methods [10, 11]. However, the diagnostic accuracy of these physical examinations has varied greatly in the literature. Test accuracy is influenced by many factors, such as swelling, reactive synovitis, and muscle guarding caused by pain in the clinical setting without anesthesia [12]. In addition, the difficulty in diagnosing partial tears has been well documented [13]. The examiners’ small hand size or the patients’ bulky leg size may also make it difficult to perform these tests and lead to false results [14, 15].

Therefore, we modified the anterior drawer test and refer to it as the “MADT.” In this test, the patient sits on the examination table with both feet hanging down freely and knees/hips flexed 90°. The examiner holds the proximal tibia and performs a push-and-pull maneuver for 2 to 3 rhythmic cycles in 1 s. Additionally, the significant laxity caused by the tibial plateau sliding anteriorly from the femoral condyles compared with that of the contralateral knees was considered positive. The aim of this study was to present the MADT and compare it with three conventional physical examinations to diagnose ACL ruptures. We expected a more sensitive and accurate detection of ACL tears. Our hypothesis was that MADT would be more accurate than other diagnostic examinations for ACL tears.

## Methods

### Patients

This study evaluated 400 consecutive patients seen at a single orthopedic outpatient department by 2 senior authors between January 2015 and September 2017. The inclusion criteria were as follows: (1) a minimum age of over 14 years old, (2) a history of knee injury or complaint of knee pain, and (3) scheduled to undergo unilateral arthroscopic surgery. The exclusion criteria were (1) a history of knee surgery, (2) fractures around the knee (ipsilateral femur, tibial, or patellar fractures), (3) bilateral knee diseases, (4) a positive medial/lateral collateral ligament stress test, and (5) proven multiligamentous injuries. All patients provided written informed consent. The study was performed according to the ethical standards of the Ethics Committee of the National Health Commission, and written approval was obtained from the Ethics Committee of Huashan Hospital Fudan University.

### Design

The performance of 4 physical maneuvers to detect ACL tears using the MADT, the anterior drawer test, the Lachman test, and the pivot shift test was prospectively evaluated. Laxity in MADT, anterior drawer test, and Lachman test and clunk in the pivot shift test were considered positive compared with those of the contralateral side; otherwise, they were considered negative. Subgroups with different degrees of laxity were not classified.

All physical examinations were independently performed by two authors who were blinded to the results of magnetic resonance imaging (MRI) for the ACL if available and prohibited attendance to arthroscopic surgeries. Disagreements were resolved by consensus, and a third author was consulted when necessary. All patients were examined twice. The first tests were conducted during the outpatient interview by the authors. Then, the second tests were performed on admission for surgery by another author.

### Physical examinations

**Fig. 1:**
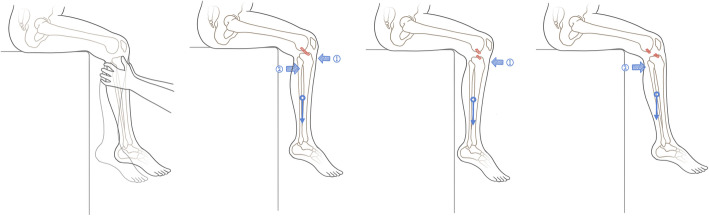
Diagram of MADT. The MADT was performed with the patients sitting on the examination table with both feet hanging down freely and knees/hips flexed 90° to relax the muscles of the knees. The examiner held the proximal tibia and do a push-and-pull maneuver with 2 to 3 rhythmic cycles in 1 s. This speed and freedom of rotating the foot enable the moment by weight of calf and foot acting on ACL as the lever fulcrum of the knee. ① the push maneuver, ② the pull maneuver

The MADT was conducted with the patients sitting on the examination table with both feet hanging down freely and knees/hips flexed 90° to relax the knee muscles. The examiner held the proximal tibia and performed a push-and-pull maneuver with 2 to 3 rhythmic cycles in 1 s (Fig. 1). This speed and freedom of rotating the foot enable the moment by weight of the calf and foot acting on the ACL as the lever fulcrum of the knee. The role of the two hands holding the proximal tibia was to elicit rhythmic anterior-posterior translation without constraining the rotation. The significant laxity caused by the tibial plateau sliding anteriorly from the femoral condyles compared with that of the contralateral knees was considered positive. This laxity was characterized by a soft anterior sliding ending point in diagnosing ACL tears, but laxity with a solid anterior sliding ending point and a soft posterior ending point was considered negative in diagnosing ACL tears, possibly indicating posterior cruciate ligament (PCL) insufficiency.

Anterior drawer tests, Lachman tests, and pivot shift tests were conducted as described in the literature [16].

### Arthroscopic surgery (golden standard)

The knee arthroscopic procedure included a thorough inspection of the ACL. The ACL tears were divided into complete, anteromedial bundle, and posterolateral bundle tears. All false-negative and positive findings were confirmed by arthroscopy in cases of isolated ACL tears.

### Statistical analysis

The indices of sensitivity, specificity, positive predictive value (PPV), negative predictive value (NPV), accuracy, diagnostic odds ratio (DOR), positive likelihood ratio (PLR), and negative likelihood ratio (NLR) were adopted to describe the diagnostic performance of the MADT. The kappa coefficient was used to assess the reproducibility of the MADT concerning interobserver variation. The area under the receiver operating characteristic curve (AUC) was calculated. The chi-squared test or Fisher’s test was adopted to evaluate the sensitivity, specificity, accuracy, PPV, and NPV, and the *z*-test was adopted to compare the AUCs of the four physical tests. A value of 0.05 was set as the level of significance. Statistical processing was conducted using the STATA 14.2 software package (STATA Inc., College Station, TX, USA).

## Results

The age of 400 patients averaged 29.74 ± 6.95 years (range, 14–49 years). A total of 296 male and 104 female patients with 218 left and 182 right involved knees were included in the study. During the diagnostic arthroscopic procedure, we found 148 (37.0%) ACL-injured knees, among which 126 (31.5%) were complete ruptures and 22 (5.5%) cases were partial tears (8 AM bundle and 14 PL bundle ruptures). There were 53 acute (≤ 3 weeks) and 95 nonacute (> 3 weeks) ACL-injured knees. The other cases comprised 191 meniscal tears, 36 cases of synovitis, and 25 cases with miscellaneous diseases (Table 1).

**Table 1:** Basic demographic and epidemiologic data

Variables (subgroups)	Number (%)
Gender (male/female)	296/104 (74.0/26.0)
Age (mean ± SD; years)	28.7 ± 6.95
Side (left/right)	218/182 (54.5/45.6)
Arthroscopic diagnosis (ACL injury/meniscal lesion/synovitis/miscellaneous)	148/191/39/25 (37.0/47.8/9.8/5.4)
ACL injury (complete ruptures/partial tear/intact)	126/22/252 (31.5/5.5/53.0)
Presentation of ACL injury (acute/chronic)	53/95 (35.8/64.2)

The sensitivity, specificity, accuracy, PPV, NPV, PLR, NLR, DOR, and AUC for the four physical tests are listed in Table 2. The sensitivity of the MADT was significantly greater than that of the anterior drawer test (*χ*^2^ = 24.249, *p* < 0.001), Lachman test (*χ*^2^ = 4.874, *p* = 0.027), and pivot shift test (*χ*^2^ = 22.829, *p* < 0.001). The specificity of the MADT was similar to that of the anterior drawer test (*χ*^2^ = 0.033, *p* = 0.715), Lachman test (*χ*^2^ = 3.143, *p* = 0.076), and pivot shift test (*χ*^2^ = 1.094, *p* < 0.296). The accuracy of the MADT was significantly higher than that of the anterior drawer test (*χ*^*2*^ = 16.226, *p* < 0.001), Lachman test (*χ*^2^ = 8.463, *p* = 0.004), and pivot shift test (χ^2^ = 13.443, *p* < 0.001). The MADT is a statistically noninferior test compared with the anterior drawer test, Lachman test, or pivot shift test based on PPV, NPV, PLR, and NLR. The DOR of the MADT was 122.92, which is considerably increased compared with that of the anterior drawer test, Lachman test, and pivot shift test at 24.84, 32.13, and 55.45, respectively.

**Table 2:** The diagnostic values of the four tests detecting an ACL ruptures

Items	MADT	ADT	LT	PST
True-positive, *n*	131	95	117	91
True-negative, *n*	237	235	225	243
False-positive, *n*	15	17	27	9
False-negative, *n*	17	53	31	57
Sensitivity (%)^a^	88.5^a^	64.2	79.1	61.5
Specificity (%)	94	93.2	89.3	97.2
Accuracy (%)^b^	92	82.5	85.5	83.5
PPV (%)	89.7	84.8	81.3	91
NPV (%)	93.3	81.6	87.9	81
PLR	14.75	9.44	7.39	21.96
NLR	0.12	0.38	0.23	0.4
DOR^c^	122.92	24.84	32.13	55.45
AUC (mean ± SD)^d^	0.925 ± 0.014	0.787 ± 0.021	0.842 ± 0.019	0.778 ± 0.021

*PPV* positive predictive value, *NPV* negative predictive value, *PLR* positive likelihood ratio, *NLR* negative likelihood ratio, *DOR* diagnostic odds ratio, *AUC* area under the curve, *SD* standard deviation, *ADT* anterior drawer test, *LT* Lachman test, *PST* pivot shift test

^a^The sensitivity of the MADT was significantly better than ADT (*χ*^2^ = 24.249, *p* < 0.001), LT (*χ*^2^ = 4.874, *p* = 0.027), and PST (*χ*^2^ = 22.829, *p* < 0.001)

^b^The accuracy of MADT was significantly higher than ADT (*χ*^2^ = 16.226, *p* < 0.001), LT (*χ*^2^ = 8.463, *p* = 0.004), and PST (*χ*^2^ = 13.443, *p* < 0.001)

^c^The diagnosis odds ratio (DOR) of MADT was 122.92, with ADT, LT, and PST were 24.84, 32.13, and 55.45

^d^The AUC of the MADT was significantly larger than that of the ADT (*z* = 5.348, *p* < 0.001), LTs (*z* = 3.435, *p* < 0.001), and PST (*z* = 5.699, *p* < 0.001)

In general, a diagnostic test with an AUC greater than 0.8 or 0.90 was considered good or appreciable, respectively [17]. As a good description index of diagnostic tools, the AUC of the MADT was 0.92 ± 0.01 and significantly greater than that of the anterior drawer test (*z* = 5.348, *p* < 0.001), Lachman test (*z* = 3.435, *p* < 0.001), and pivot shift test (*z* = 5.699, *p* < 0.001).

The MADT had an equivalent sensitivity to detect both acute/nonacute ACL ruptures (*χ*^2^ = 1.682, *p* = 0.195) and anteromedial/posterolateral bundle tears (*p* = 0.130) (Table 3). For the detection of partial ruptures of the ACL, the sensitivity of the MADT was significantly better than that of the anterior drawer test (*p* < 0.004), Lachman test (*p* = 0.047), and pivot shift test (*p* = 0.025) (Table 4).

**Table 3:** The sensitivity of MADT for different ACL injuries

Item	Subgroup (number)	Number of positive of MADT (sensitivity)	*χ*^2^, *p* value
ACL ruptures	Acute (53)	44 (0.83)	*χ*^2^ = 1.682, *p* = 0.195
	Nonacute (95)	87 (0.92)	
Partial ACL ruptures	AM bundle (8)	6 (0.75)	*p* = 0.130 (Fisher test)
	PL bundle (14)	6 (0.43)

**Table 4:** The diagnostic sensitivity of the four tests for partial ACL ruptures

Item	Positive	Negative	*p* (Fisher test)
MADT*	12	10	N/A
ADT	3	19	0.0043
LT	6	16	0.0469
PST	5	17	0.0248

* The sensitivity of the MADT was significantly better than that of the anterior drawer test (*p < *0.004), Lachman test (*p*=0.047) and pivot shift test (*p*=0.025) in detecting partial ruptures of the ACL. *MADT*: modified anterior drawer test

In the analysis of interobserver reproducibility, the senior author reported 261 positive MADTs, and the other examiner reported 260 positive MADTs. The kappa coefficient was 0.86 for interobserver reproducibility.

## Discussion

The diagnostic performance of the widely used physical examinations to evaluate isolated ACL ruptures remains highly unstable, and in clinical settings, more accurate physical examinations for ACL ruptures are expected [10]. We proposed the “MADT” based on our clinical work to more accurately diagnose ACL tears. The MADT was not an innovative method but was somewhat slightly modified to the anterior drawer test in the sitting position. The results of this study showed that our proposed clinical test can detect ACL tears well with good sensitivity, accuracy, DOR, and AUC compared with those of the anterior drawer test, Lachman test, and pivot shift test mentioned above.

One of the advantages of MADT is that it indicates a higher sensitivity regardless of the time elapsed from injury. In a meta-analysis comparing the three physical examinations, the sensitivity of the anterior drawer test, Lachman test, and pivot shift test in acute injuries without anesthesia was 0.38, 0.81, and 0.28, respectively [12]. In our study, the sensitivity of MADT in acute injuries was 0.83, which is superior to the anterior drawer test and pivot shift test and comparable to the Lachman test. In the most recent meta-analysis by Huang et al. [10], the overall sensitivity of the anterior drawer test was 0.73 (0.69–0.76), that of the Lachman test was 0.87 (0.84–0.90), and that of the pivot shift test was 0.49 (0.43–0.55). The sensitivity of the MADT was equivalent to the best pooled sensitivity of the Lachman test in the literature.

Another advantage of MADT was noted in the detection of partial ruptures of the ACL, and the sensitivity of MADT was significantly greater than that of the anterior drawer test, Lachman test, and pivot shift test despite the ratio of partial ACL ruptures being lower than that reported in the literature [7, 18]. The MADT had equivalent sensitivity to detect anteromedial/posterolateral bundle tears. This feature may add to the importance of MADT in clinical practice in ACL injury detection. The high sensitivity of MADT in diagnosing partial ACL injury may be the result of its ability to detect rotation of the tibia with more laxity. Finally, magnetic resonance imaging (MRI) is a good but expensive noninvasive diagnostic tool. UAE can more effectively elicit knee instability [19]. Considering that the application of physical examination occurs most frequently in the outpatient setting and that the arthroscopic gold standard is already available, we did not intend to include MR and UAE data in this study. Moreover, early detection of an ACL rupture using a clinical physical test is essential to avoid expensive and invasive additional procedures.

One may argue that the position and maneuver of the MADT are quite similar to those of the anterior drawer test. However, the two tests were different in operation and philosophy. Three differences are noted between the MADT and anterior drawer test. In the anterior drawer test, the patient was in the supine position with the hip in 45° and knee in 90° of flexion and the foot stable. The anterior sliding distance of the tibia over the femoral condyle was recorded. Despite the similar position of the hip and knee flexion, the patient is seated on the examination table in the MADT, where the calf and foot act as a “pendulum” swing in a relaxed style. This position could relax the patient with some set of rehearsals before the normal examination. This difference partially explains the similar ability of MADT to detect acute and nonacute ACL ruptures in the study because acute ACL ruptures may have more resistance caused by pain. Second, the maneuver is different. The MADT is a push-and-pull maneuver performed at a speed of 2–3 cycles every second in which free swing and rotation of the foot enable the moment by weight of the calf and foot acting on the ACL as the fulcrum of the lever. However, in the anterior drawer test, instability of the knee is elicited directly by the examiner’s pull. Finally, the role of two hands holding the proximal tibia to elicit rhythmic anterior-posterior translation without constraining rotation may theoretically facilitate both AP and rotational instability caused by ACL deficiency. Considering that collateral or multiligament injuries could exaggerate the instability of the knee, especially with free rotation of the tibia, we excluded cases with collateral or multiligament injuries in the study to eliminate false positives in clinical settings despite the lack of supporting data in this study. This should be the focus when examiners use MADT to diagnose ACL ruptures. In fact, the MADT is a modified anterior drawer test.

The most important tip for conducting the MADT is that when the calf and foot sway forward fastest (perpendicular to the ground), a sudden backward pushing force is given to draw forth the instability of the femoral condyle and the tibial plateau, which is especially useful for patients with a high BMI or strong muscle. The initial forward pulling force is provided to make the “pendulum” sway at a natural frequency.

There are several major limitations that should be seriously considered; otherwise, the results could be misleading on a scientific basis. The first misleading effect of any concomitant PCL tear on the results of this test is another downside. The concomitant ligament tear of the knee will theoretically exaggerate the positive MADT reports given that the positive MADT test actually merely indicates a significant anterior translation with the knee relaxed in 90° flexion. Moreover, the diagnosis of ACL ruptures is a combination of several factors added together, including injury history and clinical examination and eventually radiologic images. Second, the prevalence of ACL injury in the study group was not the same as that in a general community population but similar to that in a group of knee arthroscopic surgery candidates at one tertiary medical center. The data could not be simply generalized to the common population given that the prevalence can influence indices, such as PPV, NPV, PLR, NLR, and DOR. Third, the recently developed “lever test” was not included in this study, and we cannot compare our test to the lever test [20]. Finally, we do not have a strict numeric threshold in diagnosing ACL ruptures because the pull-and-push maneuver cannot obtain a stable distance in the clinical setting. The MADT is not an instrumented and digitalized examination, but we found that the diagnostic criteria in the MADT can be easily practiced and reproducible. Positive MADT results were characterized by a specific soft anterior sliding ending point in diagnosing ACL tears, but laxity with a solid anterior sliding ending point and a soft posterior ending point was considered negative in diagnosing ACL tears, indicating PCL insufficiency.

In conclusion, the MADT is also an alternative diagnostic test to detect ACL tears, which is equally superior to the anterior drawer test, Lachman test, and pivot shifting test. This test could improve the diagnosis of ACL ruptures combined with other clinical information, including injury history, clinical examination, and radiological findings.

## Supplementary Information

**Additional file 1. ** How to perform the MADT?.

**Additional file 2.** Some introductions of the MADT.

## Data Availability

The datasets used and/or analyzed during the current study are available from the corresponding author on reasonable request.

## Abbreviations

ACL: Anterior cruciate ligament
AUC: Area under the curve
DOR: Diagnostic odds ratio
MADT: Modified anterior drawer test
NLR: Negative likelihood ratio
NPV: Negative predictive value
PPV: Positive predictive value
PCL: Posterior cruciate ligament
PLR: Positive likelihood ratio
